# Serum KL-6 is a predictor of outcome in pulmonary alveolar proteinosis

**DOI:** 10.1186/1750-1172-8-53

**Published:** 2013-04-04

**Authors:** Francesco Bonella, Shinichiro Ohshimo, Cai Miaotian, Matthias Griese, Josune Guzman, Ulrich Costabel

**Affiliations:** 1 Department of Pneumology/Allergy, Ruhrlandklinik, University Hospital, University of Duisburg-Essen, Essen, Germany; 2 Department of Molecular and Internal Medicine, Graduate School of Biomedical Sciences, Hiroshima University, Hiroshima, Japan; 3 Dr. von Haunersches Kinderspital, University of Munich, Munich, Germany; 4 General and Experimental Pathology, Ruhr University, Bochum, Germany

**Keywords:** Pulmonary alveolar proteinosis, Orphan disease, Prognostic biomarkers, KL-6

## Abstract

**Background:**

Pulmonary alveolar proteinosis (PAP) is a rare disorder characterised by abundant alveolar accumulation of surfactant lipoproteins. Serum levels of KL-6, high molecular weight human MUC1 mucin, are increased in the majority of patients with PAP. The prognostic significance of KL-6 in PAP is still unknown. Aim of the study was to evaluate whether serum KL-6 levels correlate with the outcome of the disease.

**Patients and methods:**

From 2006 to 2012, we prospectively studied 33 patients with primary autoimmune PAP. We measured serum KL-6 levels by ELISA (Eisai, Tokyo, Japan), and evaluated the correlation between initial KL-6 levels and clinical variables. Disease progression was defined as deterioration of symptoms, and/or lung function, and/or chest imaging.

**Main results:**

The initial serum KL-6 levels were significantly correlated with the baseline PaO_2_, A-aDO_2_, DLCO, VC and TLC (p=0.042, 0.012, 0.012, 0.02 and 0.013, respectively). The change over time of serum KL-6 correlated with the change over time of DLCO (p=0.017). The initial serum KL-6 levels were significantly higher in patients with disease progression than in those with remission (p<0.001). At a cut-off level of 1526 U/mL, the initial serum KL-6 level predicted disease progression (Se 81%, Sp 94%). At a cut-off level of 2157 U/mL, the initial serum KL-6 predicted the necessity of repeated whole lung lavage (Se 83%, Sp 96%). In the multivariate analysis, the initial serum level of KL-6 was the strongest predictor of disease progression (HR 9.41, p=0.008).

**Conclusions:**

Serum KL-6 seems to predict outcome in PAP.

## Background

Pulmonary alveolar proteinosis (PAP), first described in 1958 [1], is a rare syndrome characterized by the intra-alveolar accumulation of surfactant lipids and proteins. PAP can occur in three distinct clinical forms: hereditary (caused by mutations in the GM-CSF receptor gene), primary autoimmune (associated with GM-CSF autoantibodies), and secondary to several underlying conditions (malignancies, toxic agents, immunosuppression) [2-6].

KL-6 is a mucin-like glycoprotein present in the human MUC1 mucin [7]. The origin of the elevated KL-6 in PAP has been shown to be the hyperplastic alveolar type II pneumocyte [8]. After production, KL-6 has been found to be immunolocalized in the fine granular substance of the alveoli, in a distribution that is similar to SP-A [9]. It has been hypothesized that KL-6 is transferred into the bloodstream via lymphatic vessels [8]. KL-6 concentrations in BAL are 3–5 fold higher than in serum of PAP patients [10,11].

The role of serum KL-6 as sensitive biomarker for various interstitial lung diseases has been demonstrated in idiopathic pulmonary fibrosis, radiation pneumonitis, drug-induced pneumonitis, hypersensitivity pneumonitis, CTD-associated ILD, pulmonary sarcoidosis, and cystic fibrosis [12-15]. Serial changes of serum KL-6 predict the short-term prognosis in rapidly progressive IPF [16], and initial serum KL-6 levels are associated with long-term survival in IPF and pulmonary fibrosis in connective-tissue disease [17-21].

The clinical utility of serum KL-6 in PAP has been only partially investigated [10,22-25]. The aim of the present study was to evaluate the prognostic utility of serum KL-6 level in a relevant cohort of PAP patients. Some of the results of this study have been previously reported in the form of an abstract [26].

## Patients and methods

### Disposition of the patients

We prospectively studied 33 Caucasian PAP patients between 2008 and 2012. The study was approved by the local IRB (approval number 06–3170). Informed consent was obtained from the patients.

### Diagnosis of PAP

The diagnosis of PAP was based on diagnostic BAL findings, characteristic HRCT, and/or histopathologic findings on biopsy [4,27]. All the patients had the primary autoimmune form: GM-CSF autoantibodies were detected in all patients (Table 1).

**Table 1:** Baseline demographics and patients’ characteristics of all patients and of subgroups according to disease outcome (all data collected at baseline)

		**Outcome**		
	**All patients**	**Remission**	**Progression**	**p**
	**N=33**	N=17 (51) *	**N=16 (49)**	
**Gender** (M/F) N(%)	18/15 (54/46)	6/11	12/4	0.016†
**Age** (yrs)	49±12 (19–79)	50±9	47±15	n.s.
**Smoking habits** (never/ex/current) N(%)	5/12/16 (15/36/49)	5/5/7	0/7/9	0.055†
**BMI** (kg/m^2^)	26±5 (19–36)	28±4	25±5	n.s.
**PaO2** (mmHg)	72±15 (47–117)	78±13	63±12	0.002
**A-aDO2** (mmHg)	37±14 (11–63)	31±11	44±14	0.005
**VC** (% pred)	80±16 (44–123)	88±13	69±13	0.001
**FEV1** (% pred)	75±16 (43–104)	80±15	67±13	0.014
**TLC** (% pred)	79±17 (42–116)	85±16	70±14	0.009
**DLCO** (% pred)	57±19 (21–96)	66±12	42±13	0.001
**GM-CSF autoantibody** (μg/mL) ^§^	48±22	60±17	44±13	0.55
**KL-6** (U/mL)	2049±1893	1084±585	3334±2267	0.001
**LDH** (IU/L) ^§^	283±93 (140–522)	243±69	338±103	0.005
**CEA** (ng/mL) ^§^	8.5±7 (1–27)	6±4.5	12±5.5	0.048

Unless otherwise indicated, values are expressed as mean ± SD (range).

*n.s.*= not significant.

* patients who improved or remained stable and did not receive WLL within 18 months prior to the last evaluation.

§ the cut-off of normality for each biomarker is reported in the methods.

† Fisher's exact test, for all other comparisons in the table Student's t-test was used.

### Definition of disease progression, improvement and remission

Disease progression was defined as deterioration of symptoms (worsening of dyspnea, cough, chest pain and weight loss), and/or lung function (decrease in forced vital capacity >10%), and/or chest imaging (increase of the previous findings or appearance of new infiltrates characteristic of PAP) since the last follow-up visit. In order to avoid the bias of subjective interpretation in the assessment of disease progression, we included in this group with disease progression only the patients who required treatment with whole lung lavage (WLL) in the follow-up period. In our centre, PAP patients should be submitted to WLL (1) in presence of persistent (PaO2 <70 mmHg with no change for at least 3 months) or progressive respiratory failure (decrease in PaO2 >10 mmHg from the last follow up visit, or necessity of oxygen treatment in the last 3 months); (2) in the absence of respiratory difficulty at rest, the presence of exercise desaturation (decrease in PaO2 >10 mmHg or SaO2 >5%).

We defined a patient as improved if PAP symptoms (dyspnea, cough, chest pain and weight loss) ameliorated or disappeared and lung function tests improved (forced vital capacity >5% and/or DLCO >10% and/or PaO_2_ > 10 mmHg since the last measurement), and chest imaging showed stability or amelioration of the previous findings.

A stable course of disease was defined as absence of new PAP symptoms or no worsening of the previous symptoms (see above), no change in lung function tests and no new radiological infiltrates. We considered a patient as stabilized if the disease course changed from progressive to stable (see the definition above) after treatment or spontaneously.

A patient was defined as being in remission, if he/she improved or remained stable and did not receive WLL within 18 months prior to the last evaluation. The 18 months limit is based on longterm experience in our centre.

### KL-6, GM-CSF autoantibody and other laboratory assays

Serum samples were obtained by venipuncture at time of first evaluation and then every 202±148 days, and were stored at −20 or −80°C until analysis.

Serum KL-6 was measured by ELISA (Eisai Co. Ltd., Tokyo, Japan) as described before [10] (upper limit of normal <458 U/mL as determined in 142 Caucasian healthy subjects).

GM-CSF autoantibody concentration was measured by ELISA as previously reported [28-30]. GM-CSF values <10 μg/mL are to be considered as normal [31].

CEA and LDH were routinely measured in serum (normal value for CEA <2.5 ng/mL and for LDH <225 IU/L).

### Pulmonary function tests

Measurements including vital capacity (VC), forced expiratory volume in one second (FEV1), total lung capacity (TLC), diffusing capacity of the lung for carbon monoxide (DLCO), partial pressure of oxygen in arterial blood (PaO2), and alveolar-arterial oxygen gradient (A-aDO2) were performed along with the blood sample collection. Values were expressed as percentages of predicted normal values [32].

### Statistics

Continuous variables were evaluated for a normal distribution with the Kolmogorov-Smirnov test. Parametric data are presented as mean ± SE and nonparametric data are presented as median and interquartile range (IQR). Categorical variables are presented as either a percentage of the total or numerically, as appropriate. Comparison between 2 groups was done with Student’s *t*-test or Wilcoxon’s rank test for continuous variables, Chi-squared or Fischer’s exact test for categorical variables. Spearman’s or Pearson’s correlation coefficient was obtained for correlations. Receiving operating curves (ROC) analysis was used to predict disease outcome. Uni and Multivariate Cox’s proportional hazard regression model was used to analyze prognostic factors. The Kaplan-Meier method with log-rank test was used to analyze whether KL-6 levels were associated with the disease outcome. *P* values of <0.05 were considered statistically significant. All statistical analyses were performed using SPSS 17 (SPSS Inc., Chicago, IL, USA).

## Results

### Demographics and patients´ outcome

33 PAP patients were prospectively studied. The median follow-up time was 510 (90–1890) days. Demographics and disease outcome of the patients are shown in Table 1. GM-CSF autoantibodies were detected in all 33 PAP patients. All patients experiencing disease progression (n=16) were treated with whole lung lavage (WLL). A subgroup of patients (n=12) needed repeated WLL during the follow-up before they stabilized. 17 patients reached remission. At baseline, 21 patients had already received at least one WLL before the first evaluation. Of them, only 5 patients, all referred from other hospitals, had received a WLL within 18 months prior to the first evaluation.

### Serum levels of KL-6

Mean serum KL-6 level was 2049±1893 U/mL (Table 1). Men had higher KL-6 serum levels than women (2729±2311 vs 1240±656 U/mL, p=0.018). No differences in KL-6 serum levels were seen according smoking habits or fume/dust exposure (data not shown). No correlations were seen between KL-6 serum levels and age or BMI (data not shown).

**Figure 1:**
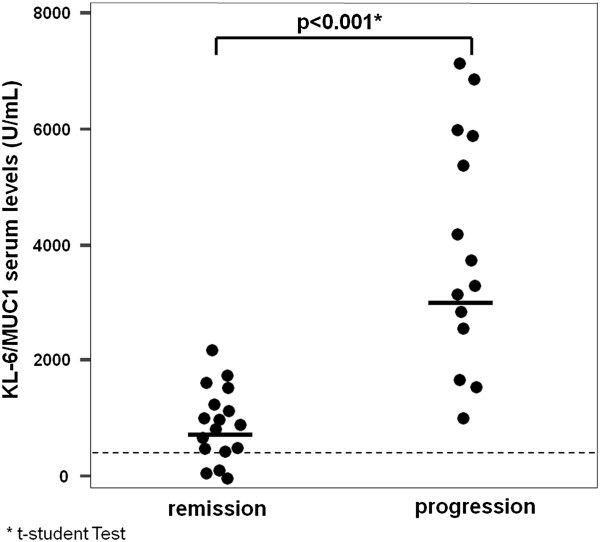
**Initial KL-6 serum levels and disease outcome.** The graph shows the initial KL-6 serum concentrations in patients having progression or remission of disease during the follow-up. Dots represent single patients. Grid line represents the upper limit of normal (<458 U/mL). Bold lines represent the mean value.

Patients with disease progression had higher initial serum KL-6 levels than patients with improved/stable disease (3334±2267 vs 1084±585 U/mL, p<0.001) (Figure 1).

**Figure 2:**
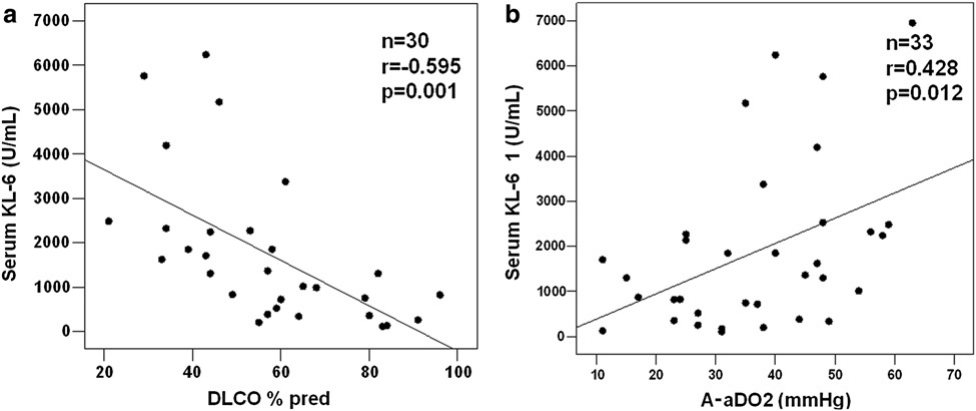
**Correlation between initial KL-6 serum levels and pulmonary diffusing capacity.** The graph shows the correlation between initial KL-6 serum levels and (**a**) DLCO, (**b**) A-aDO2.

Initial KL-6 serum levels correlated directly with A-aDO2 (r=0.428, p=0.012), inversely with PaO2 (r=−0.35, p=0.042), FVC (r=−0.41, p=0.02), TLC (r=−0.421, p=0.013) and DLCO (r=−0.595, p=0.001). The strongest correlations are shown in Figure 2. No correlations were seen between initial serum KL-6 and initial serum LDH or GM-CSF autoantibody (data not shown).

### Changes in KL-6 serum levels over time

**Figure 3:**
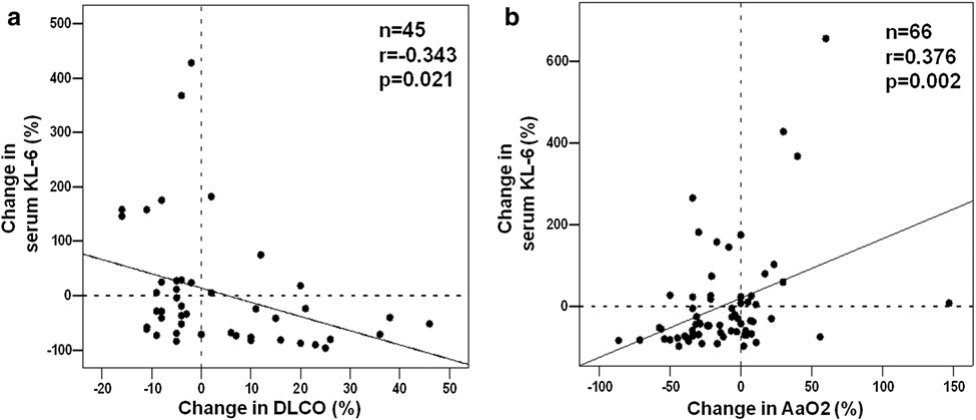
**Correlation between change in KL-6 serum levels and pulmonary diffusing capacity over time.** The graph shows the correlation between initial KL-6 serum levels and change in (**a**) DLCO and (**b**) A-aDO2 over time. Shown are % values (=relative change from baseline).

Correlations between changes in serum KL-6 levels and changes in pulmonary function tests (PFTs) are shown in Figure 3. Patients whose DLCO improved during the follow-up period had decreasing serum KL-6 concentrations. In these patients, the change in KL-6 production was expressed as a negative value, because serum KL-6 concentrations were higher at the beginning than at the end of the observation period.

### Predictive value of baseline serum KL-6 levels for the outcome of PAP

**Figure 4:**
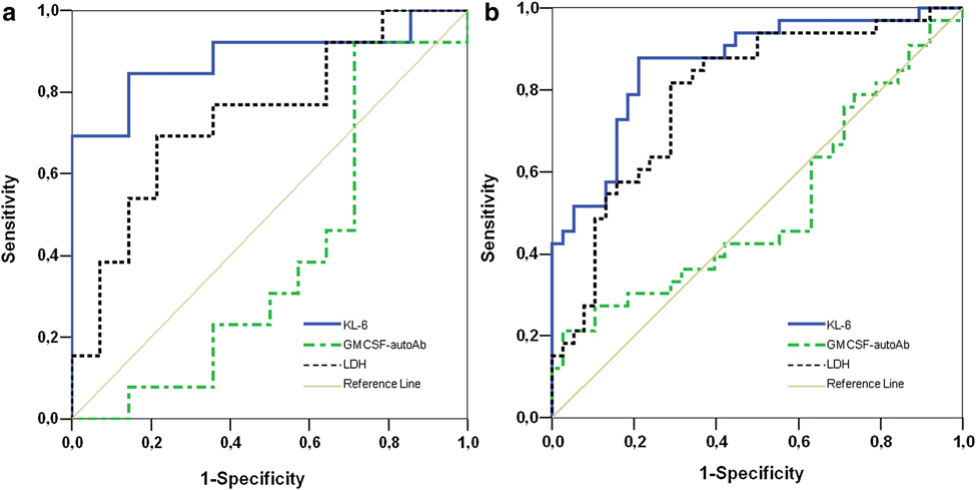
**Receiver operating characteristic curve analysis.** The curves show the power of initial serum KL-6, LDH and GM-SCF for predicting (**a**) disease progression, and (**b**) necessity of repeated WLL.

ROC analysis was performed to test whether baseline serum KL-6 concentrations were predictive of disease progression, the necessity of repeated WLL, or remission. Baseline serum KL-6 concentrations were associated with disease progression and the necessity of repeated WLL (Figure 4). ROC analysis for Serum LDH and GM-CSF autoantibody did not show a predictive value for disease progression (p=0.06 and p=0.46, respectively). Therefore, we proceeded with the cut-off calculation only for serum KL-6.

At the cut-off level of ≥1526 U/mL, serum KL-6 levels yielded a sensitivity of 81% and a specificity of 94% to predict disease progression (AUC=0.87, p=0.001). A second cut-off was identified at ≥2157 U/mL with a sensitivity of 83% and a specificity of 96% to predict the necessity of repeated WLL (p<0.0001). PPV and NPV and accuracy are reported in Table 2.

**Table 2:** Prognostic value of serum KL-6 for disease progression and for necessity of treatment with repeated WLL

	**Se (%)**	**Sp (%)**	**PPV**	**NPV**	**Accuracy**
**KL-6 ≥1526 U/mL**					
Disease progression	81	94	93	84	88
**KL-6 ≥2157 U/mL**					
Necessity of repeated WLL	83	96	91	92	94

Se=sensitivity, *Sp*=specificity, *PPV*= positive predictive value, *NPV*= negative predictive value.

To verify the utility of the cut-off for disease progression, we divided all patients into two groups; the high KL-6 group (n=14), baseline concentrations ≥1526 U/mL) and the low KL-6 group (n=19), baseline concentrations <1526 U/mL). The characteristics of the stratified patients according to the KL-6 cut-off are shown in Table 3.

**Table 3:** Characteristics of the patients stratified according to the KL-6 predictive cut-off for disease progression (N=33)

	**KL-6 <1526 U/mL**	**KL-6 ≥1526 U/mL**	**p**
	**(N or mean ± SE)**	**(N or mean ± SE)**	
**Gender** (M/F)	9/10	9/5	ns*
**Age** (yrs)	52±10	46±11	ns**
**Smoking history** (yes/no)	15/4	12/2	ns*
**Clinical course**			
-PAP-related death (yes/no)	0/19	2/12	ns*
-disease progression (yes/no)	3/16	13/1	<0.0001*
**Treatment** (yes/no)	6/13	12/2	0.03*
-cumulative number of WLL	3.5±3	7±6.5	0.06
**PaO2** (mmHg)	78±14	66±12	0.012**
**A-aDO2** (mmHg)	31±12	42±14	0.023**
**VC** (% pred)	88±15	72±14	0.002**
**FEV1** (% pred)	81±15	67±14	0.009**
**TLC** (% pred)	86±17	72±16	0.017**
**DLCO** (% pred)	70±15	42±12	<0.0001**
**GM-CSF autoantibody** (μg/mL)	44±19	55±13	ns**
**KL-6** (U/mL)	930±352	3934±1756	<0.0001**
**LDH** (IU/L)	244±78	327±109	ns**
**CEA** (ng/mL)	5.5±3.8	7.5±6	ns**

Unless otherwise indicated, values are expressed as mean ± SD (range).

*ns*= not significant.

* Fischer´ s exact test.

**Student´ s *t*-test.

**Figure 5:**
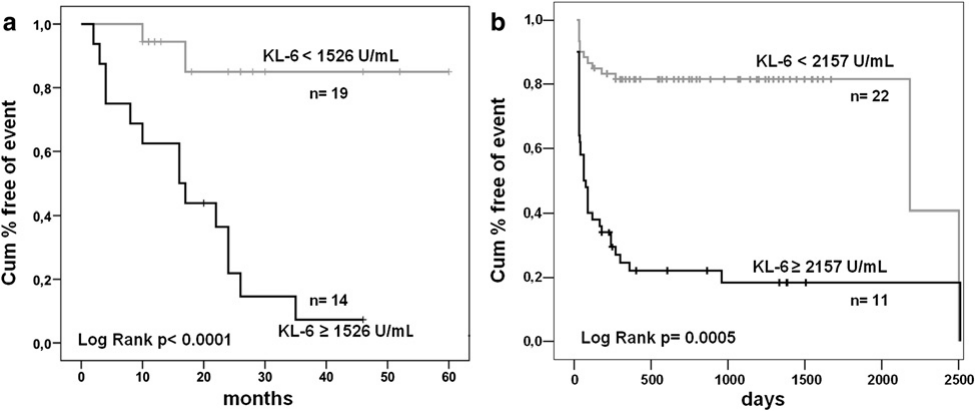
**Kaplan-Meier analysis.** The graph shows the predictive value of initial serum KL-6 levels for (**a**) disease progression and (**b**) the necessity of repeated WLL.

Plots of the incidence of disease progression and treatment with repeated WLL for both groups were obtained using the Kaplan-Meier curve (Figure 5). The incidence of disease progression was 80% in the high KL-6 group (n=14) and 15% in the low KL-6 group (n=19) (log-rank test, p<0.0001). The incidence of treatment with at least one WLL was significantly greater in the high KL-6 group than in the low KL-6 group (73% vs 30%, p=0.028).

### Predictive factors for the outcome of PAP evaluated by the Cox proportional hazard models

We explored the predictive value of KL-6 for disease progression. In the univariate model, baseline serum KL-6 levels ≥1526 U/mL were associated with an increased risk of disease progression (HR, 6.28; 95% CI, 1.77-22.35; p=0.005) (Table 4). In the multivariate model taking into account serum markers, baseline demographics and pulmonary function variables (Table 5), serum KL-6 levels ≥1526 U/mL remained predictive for disease progression when data were adjusted for covariates (HR, 3.84; 95% CI, 0.46-288.12; p=0.046).

**Table 4:** Univariate Cox proportional hazard model evaluating predictors for disease progression and the necessity of repeated WLL

**Variable**	**Hazard ratio**	**CI 95%**		**p value**
**Disease progression**				
**KL-6** U/mL (cont.)	1.000	1.000	1.001	0.002
**KL-6 ≥1526** U/mL (binary)	6.284	1.767	22.352	0.005
**Age**, yrs (cont.)	0.967	0.922	1.010	0.164
**Gender** (female=1)	5.448	1.509	19.671	0.010
**Smoking history** (positive=1)	27.203	0.087	8539.00	0.260
**LDH** IU/L (cont.)	1.007	1.002	1.012	0.003
**GM-CSF autoantibodies** μg/mL (cont.)	0.993	0.968	1.020	0.617
**PaO2,** mmHg (cont.)	0.935	0.892	0.980	0.005
**A-aDO2,** mmHg (cont.)	1.067	1.020	1.115	0.010
**VC,** % pred. (cont.)	0.955	0.922	0.989	0.009
**DLCO,** % pred. (cont.)	0.948	0.909	0.989	0.012
**Necessity of repeated WLL**				
**KL-6** U/mL (cont.)	1.000	1.000	1.000	<0.001
**KL-6 ≥2157** U/mL (binary)	20.776	4.316	100.015	<0.0001
**Age**, yrs (cont.)	0.934	0.883	0.989	0.019
**Gender** (female=1)	2.910	0.786	10.769	0.110
**Smoking history** (positive=1)	28.329	0.060	13470.548	0.288
**LDH,** IU/L (cont.)	1.010	1.004	1.017	0.001
**GM-CSF autoantibodies,** μg/mL (cont.)	0.996	0.967	1.025	0.767
**PaO2,** mmHg (cont.)	0.923	0.875	0.975	0.004
**A-aDO2,** mmHg (cont.)	1.095	1.032	1.162	0.003
**VC,** % pred. (cont.)	0.940	0.906	0.975	0.001
**DLCO,** % pred. (cont.)	0.935	0.892	0.980	0.005

Definition of abbreviation: *CI*; confidence interval.

**Table 5:** Multivariate Cox proportional hazard model evaluating predictors for disease progression and the necessity of repeated WLL

**Variable**	Hazard ratio*	**CI 95%**		**p value**
**Disease progression**				
**KL-6,** U/mL (cont.)	0.999	0.998	1.000	0.090
**KL-6 ≥1526,** U/mL (binary)	3.844	0.460	288.120	0.046
Gender (female=1) (binary)	18.030	2.378	136.696	0.051
**Necessity of repeated WLL**				
**KL-6,** U/mL (cont.)	1.000	1.000	1.001	0.196
PaO2, mmHg (cont.)	0.897	0.579	0.969	0.028
**KL-6 ≥2157,** U/mL (binary)	9.408	0.794	111.511	0.008
PaO2, mmHg (cont.)	0.97	0.93	1.00	0.038
LDH, IU/L (cont)	46.449	1.661	1299,262	0.024

Definition of abbreviation: *CI*; confidence interval.

* Calculated by stepwise conditional LR method. All significant predicting factors in the univariate analysis were included in the multivariate model. Only the factors that influence the model (adjusting factors) are reported in the Table.

We also explored the predictive value of baseline serum KL-6 levels ≥2157 U/mL for the necessity of treatment with repeated WLL (a subgroup of 12 patients) (HR, 20.77; 95% CI, 4.32-100.01; p<0.0001) (Table 4). In the multivariate model baseline serum KL-6 levels ≥2157 U/mL remained predictive for treatment with repeated WLL when data were adjusted for covariates (HR, 9.408; 95% CI, 0.794-111.511 p=0.008) (Table 5).

Interestingly, the combination of serum KL-6 ≥2157 U/mL and serum LDH reached a stronger predictive value for the treatment with repeated WLL (HR 46.45, 95% CI, 1.66-1299.26; p=0.024) than serum KL-6 alone. However, serum LDH alone did not show a predictive value for repeated WLL (HR 1.03, 95% CI, 0.99-1.06; p=0.068), confirming the results of ROC analysis (see results above).

## Discussion

This study showed that serum KL-6 levels correlate with disease progression and can predict outcome in a cohort of adult European PAP patients. At a cut-off value of 1526 U/mL for serum KL-6, the sensitivity and specificity to predict disease progression was 81% and 94%, respectively. The cut-off value of 2157 U/mL had a sensitivity of 83% and a specificity of 96% to identify patients that needed repeated WLL. Moreover, multivariate regression analysis confirmed serum KL-6 as the strongest predicting factor for disease progression and repeated WLL.

It is known that serum KL-6 levels are extremely elevated in PAP patients, with mean values being much higher than those in any other ILD [3,12,20,23,33,34]. In our PAP cohort the mean serum KL-6 level (mean, 2049 U/mL) was considerably lower than reported by Inoue et al. in Japanese PAP patients (mean, 6067 U/mL) [3], but similar to the value observed in Chinese PAP patients (mean, 3127 U/mL) [11]. The prevalence of a particular genetic variant in the MUC1 gene may influence the serum levels of KL-6, as previously reported in a Dutch cohort [34].

We confirmed published data on the association of serum KL-6 with lung function tests and blood gas analyses [3,10,11]. Patients with progressive disease had higher levels of serum KL-6, as described by Inoue et al. [3]; the correlation with DLCO and PaO2 was confirmed in our patients [10,11].

Whereas a serum KL-6 level ≥1526 U/mL predicted disease progression with a sensitivity of 81% and a specificity of 94% neither serum LDH or serum GM-CSF autoantibody did show a predictive value for disease progression. The poor correlation with severity of disease and the weak power of GM-CSF autoantibodies to predict PAP outcome are known [3,10,14,30,35,36]. The better predictive value of serum KL-6 in comparison to serum GM-CSF autoantibody may be explained by the origin and the metabolism of KL-6. Whereas GM-CSF autoantibodies are produced systemically by B-cell derived plasmocytes, KL-6 is locally produced by pneumocytes type II and then accumulates in the proteinaceous material of PAP patients [9,24]. KL-6 is consistently present in the epithelial lining fluid in PAP and various ILDs [24,37]. The mechanisms of increase in serum KL-6 level are thought to be an increase in KL-6 production and/or an increased permeability of the air– blood barrier in the affected lungs [13,20].

The patients with serum KL-6 levels ≥1526 U/mL had worse lung function tests than patients with lower levels, but the serum levels of LDH, GM-CSF autoantibodies and CEA did not differ significantly between the two groups. The incidence of disease progression was 80% in patients with higher serum KL-6 levels versus 15% in those with lower levels. The multivariate analysis showed that a serum KL-6 level ≥1526 U/mL, but not KL-6 as continuous variable, was associated with a 3.8 hazard of disease progression even after adjusting for covariates. The threshold of 1526 U/mL for serum KL-6 established by this study might be different in other cohorts. However, this does not impact our finding that patients with high KL-6 serum concentrations have a higher risk of disease progression than patients with lower serum levels.

From previous studies it is known that 5 to 10% of PAP patients can reach a spontaneous remission [2,3,30], 75 to 90% after treatment with at least one WLL. There is a consistent subgroup of patients that need repeated WLL, above all smokers. Therefore, we investigated whether this subgroup of 12 patients can be identified at baseline. We found that a cut-off value of 2157 U/mL for initial serum KL-6 had an accuracy of 94% in identifying these patients. The high specificity of this cut-off (96%) may be helpful to identify responders to WLL. In order to test the possible role of KL-6 as a predictor for the need of repeated WLL, we performed uni- and multivariate analysis adjusting the model for covariates (age, gender and smoking habits). KL-6 at the cut-off 2157 U/mL was the strongest predictor for the need of repeated WLL, with a hazard ratio of 9.4 in the multivariate analysis.

According to our findings, the predictive power of serum KL-6 is high. Both disease progression and the need of repeated WLL are affected by patient age, gender, smoking history, and baseline physiology, but hazard proportional ratios for these parameters are below 2.0 and often not significant. In recent years, there was increasing interest to define accurate predictors of outcome in PAP. Up to now, the best predictors of survival have been PFTs or composite (clinical-physiological) parameters, like the disease severity score DSS [3,30,38]. This is the first study to demonstrate that a single serum biomarker at baseline can predict outcome in PAP.

There are several limitations of this study that need to be addressed. First, we did not check for the mucin-1 568 A/G polymorphism that is known to influence KL-6 serum levels [20,34]. In fact, even if higher levels of serum KL-6 have been reported in healthy Europeans [33,34] in comparison to Japanese, our PAP patients had lower levels of serum KL-6 than the Japanese, also with advanced disease. A validation study is needed to clarify this point. Second, we could not find a predictive value of serum KL-6 for mortality because the death rate was low in our cohort during the observational period. Third, baseline data of our patients were not absolute baseline data at time of diagnosis, but data obtained at the time of first blood sampling for serum KL-6, making the cohort at baseline remarkably heterogeneous.

In conclusion, we were able to demonstrate that KL-6 is a predictive serum biomarker for the outcome of PAP. This may be of benefit in the clinical management of patients with PAP. Although our data are promising, a multicentre validation is necessary to determine whether serum KL-6 should be routinely used as a prognostic biomarker in PAP.

### Ethical standards

The experiments in this study comply with the current laws in Germany.

## Abbreviations

A-aDO2: Alveolar arterial oxygen gradient; BALF: Bronchoalveolar lavage fluid; CTD: Connective tissue disease; DLCO: Diffusing capacity of the lung for carbon monoxide; ELISA: Enzyme-linked immunosorbent assay; FEV1: Forced expiratory volume in one second; GM-CSF: Granulocyte macrophage colony stimulating factor; HRCT: High resolution computed tomography; ILD: Interstitial lung disease; PAP: Pulmonary alveolar proteinosis; PFTs: Pulmonary function tests; ROC: Receiver operating characteristic; TBB: Trans bronchial biopsy; TLC: Total lung capacity; VC: Vital capacity; WLL: Whole lung lavage.

## Competing interests

The authors declare that they have no competing interests.

## Authors’ contributions

FB contributed to the conception and design of the study; collecting sample, performing biomarkers measurement, collecting, analyzing, and interpreting the data; and drafting the manuscript. SO contributed to analyzing and interpreting the data. MG contributed to measuring the biomarkers in serum and interpreting the data. MC contributed to measuring the biomarkers in serum, analyzing and interpreting the data. JG contributed to the conception and design of the study; and drafting the manuscript. UC contributed to the conception and design of the study; interpreting the data and drafting the manuscript, and he is the guarantor of the manuscript. All authors have read and approved the final manuscript.

## References

[B1] Rosen SH Castleman B Liebow AA **Pulmonary alveolar proteinosis** N Engl J Med 1958 258 1123 1142 10.1056/NEJM195806052582301 13552931

[B2] Seymour JF Presneill JJ **Pulmonary alveolar proteinosis: progress in the first 44 years** Am J Respir Crit Care Med 2002 166 215 235 10.1164/rccm.2109105 12119235

[B3] Inoue Y Trapnell BC Tazawa R Arai T Takada T Hizawa N Kasahara Y Tatsumi K Hojo M Ichiwata T **Characteristics of a large cohort of patients with autoimmune pulmonary alveolar proteinosis in Japan** Am J Respir Crit Care Med 2008 177 752 762 10.1164/rccm.200708-1271OC 18202348 PMC2720118

[B4] Carey B Trapnell BC **The molecular basis of pulmonary alveolar proteinosis** Clin Immunol 2010 135 223 235 10.1016/j.clim.2010.02.017 20338813 PMC2866141

[B5] Kitamura T Tanaka N Watanabe J Uchida K Kanegasaki S Yamada Y Nakata K **Idiopathic pulmonary alveolar proteinosis as an autoimmune disease with neutralizing antibody against granulocyte/macrophage colony-stimulating factor** J Exp Med 1999 190 875 880 10.1084/jem.190.6.875 10499925 PMC2195627

[B6] Costabel U Guzman J **Pulmonary alveolar proteinosis: a new autoimmune disease** Sarcoidosis Vasc Diffuse Lung Dis 2005 22 Suppl 1 67S 73S 16457018

[B7] Stahel RA Gilks WR Lehmann HP Schenker T **Third International Workshop on Lung Tumor and Differentiation Antigens: overview of the results of the central data analysis** Int J Canc Suppl 1994 8 6 26 10.1002/ijc.2910570704 8194898

[B8] Ohtsuki Y Kobayashi M Yoshida S Kishimoto N Kubo K Yokoyama A Lee GH Furihata M **Immunohistochemical localisation of surfactant proteins A and D, and KL-6 in pulmonary alveolar proteinosis** Pathology 2008 40 536 539 10.1080/00313020802198077 18604747

[B9] Kobayashi M Takeuchi T Ohtsuki Y **Differences in the immunolocalization of surfactant protein (SP)-A, SP-D, and KL-6 in pulmonary alveolar proteinosis** Pathol Int 2008 58 203 207 10.1111/j.1440-1827.2007.02212.x 18251786

[B10] Takahashi T Munakata M Suzuki I Kawakami Y **Serum and bronchoalveolar fluid KL-6 levels in patients with pulmonary alveolar proteinosis** Am J Respir Crit Care Med 1998 158 1294 1298 10.1164/ajrccm.158.4.9712003 9769294

[B11] Lin FC Chen YC Chang SC **Clinical importance of bronchoalveolar lavage fluid and blood cytokines, surfactant protein D, and Kerbs von Lungren 6 antigen in idiopathic pulmonary alveolar proteinosis** Mayo Clin Proc 2008 83 1344 1349 10.4065/83.12.1344 19046553

[B12] Kohno N Kyoizumi S Awaya Y Fukuhara H Yamakido M Akiyama M **New serum indicator of interstitial pneumonitis activity. Sialylated carbohydrate antigen KL-6** Chest 1989 96 68 73 2661160 10.1378/chest.96.1.68

[B13] Ohshimo S Bonella F Sommerwerck U Teschler H Kamler M Jakob HG Kohno N Guzman J Costabel U **Comparison of serum KL-6 versus bronchoalveolar lavage neutrophilia for the diagnosis of bronchiolitis obliterans in lung transplantation** J Heart Lung Transplant 2011 30 1374 1380 10.1016/j.healun.2011.07.010 21871820

[B14] Ohnishi H Yokoyama A Yasuhara Y Watanabe A Naka T Hamada H Abe M Nishimura K Higaki J Ikezoe J Kohno N **Circulating KL-6 levels in patients with drug induced pneumonitis** Thorax 2003 58 872 875 10.1136/thorax.58.10.872 14514942 PMC1746480

[B15] Ohshimo S Bonella F Grammann N Starke K Cui A Bauer PC Teschler H Kohno N Guzman J Costabel U **Serum KL-6 as a novel disease marker in adolescent and adult cystic fibrosis** Sarcoidosis Vasc Diffuse Lung Dis 2009 26 47 53 19960788

[B16] Yokoyama A Kohno N Hamada H Sakatani M Ueda E Kondo K Hirasawa Y Hiwada K **Circulating KL-6 predicts the outcome of rapidly progressive idiopathic pulmonary fibrosis** Am J Respir Crit Care Med 1998 158 1680 1684 10.1164/ajrccm.158.5.9803115 9817725

[B17] Yokoyama A Kondo K Nakajima M Matsushima T Takahashi T Nishimura M Bando M Sugiyama Y Totani Y Ishizaki T **Prognostic value of circulating KL-6 in idiopathic pulmonary fibrosis** Respirology 2006 11 164 168 10.1111/j.1440-1843.2006.00834.x 16548901

[B18] Satoh H Kurishima K Ishikawa H Ohtsuka M **Increased levels of KL-6 and subsequent mortality in patients with interstitial lung diseases** J Intern Med 2006 260 429 434 10.1111/j.1365-2796.2006.01704.x 17040248

[B19] Tazawa R Hamano E Arai T Ohta H Ishimoto O Uchida K Watanabe M Saito J Takeshita M Hirabayashi Y **Granulocyte-macrophage colony-stimulating factor and lung immunity in pulmonary alveolar proteinosis** Am J Respir Crit Care Med 2005 171 1142 1149 10.1164/rccm.200406-716OC 15735059

[B20] Ishikawa N Hattori N Yokoyama A Kohno N **Utility of KL-6/MUC1 in the clinical management of interstitial lung diseases** Respir Investig 2012 50 3 13 22554854 10.1016/j.resinv.2012.02.001

[B21] Bonella F Volpe A Caramaschi P Nava C Ferrari P Schenk K Ohshimo S Costabel U Ferrari M **Surfactant protein D and KL-6 serum levels in systemic sclerosis: correlation with lung and systemic involvement** Sarcoidosis Vasc Diffuse Lung Dis 2011 28 27 33 21796888

[B22] Lin FC Chen YC Chang SC **Clinical importance of bronchoalveolar lavage fluid and blood cytokines, surfactant protein D, and Kerbs von Lungren 6 antigen in idiopathic pulmonary alveolar proteinosis** Mayo Clin Proc 2008 83 1344 1349.23 10.4065/83.12.1344 19046553

[B23] Nakajima M Manabe T Niki Y Matsushima T **Serum KL-6 level as a monitoring marker in a patient with pulmonary alveolar proteinosis** Thorax 1998 53 809 811 10.1136/thx.53.9.809 10319067 PMC1745314

[B24] Ishikawa N Kondo K Oguri T Kamitsuna M Sakurai J Fujitaka K Yamasaki M Maeda H Isobe T Kohno N **Usefulness of the modified lavage technique of Bingisser and KL-6 monitoring in a patient with pulmonary alveolar proteinosis** Intern Med 2002 41 381 385 10.2169/internalmedicine.41.381 12058888

[B25] Fujishima T Honda Y Shijubo N Takahashi H Abe S **Increased carcinoembryonic antigen concentrations in sera and bronchoalveolar lavage fluids of patients with pulmonary alveolar proteinosis** Respiration 1995 62 317 321 10.1159/000196473 8552862

[B26] Ohshimo S Bonella F Cai MT Horimasu Y Sarría R Guzman J Costabel U **Serum KL-6 As Predictor Of Disease Progression In Patients With Pulmonary Alveolar Proteinosis** Am J Respir Crit Care Med 2011 183 A1619

[B27] Costabel U Guzman J Bonella F Oshimo S **Bronchoalveolar lavage in other interstitial lung diseases** Semin Respir Crit Care Med 2007 28 514 524 10.1055/s-2007-991525 17975779

[B28] Latzin P Tredano M Wust Y de Blic J Nicolai T Bewig B Stanzel F Kohler D Bahuau M Griese M **Anti-GM-CSF antibodies in paediatric pulmonary alveolar proteinosis** Thorax 2005 60 39 44 10.1136/thx.2004.021329 15618581 PMC1747161

[B29] Kitamura T Uchida K Tanaka N Tsuchiya T Watanabe J Yamada Y Hanaoka K Seymour JF Schoch OD Doyle I **Serological diagnosis of idiopathic pulmonary alveolar proteinosis** Am J Respir Crit Care Med 2000 162 658 662 10.1164/ajrccm.162.2.9910032 10934102

[B30] Bonella F Bauer PC Griese M Ohshimo S Guzman J Costabel U **Pulmonary alveolar proteinosis: new insights from a single-center cohort of 70 patients** Respir Med 2011 105 1908 1916 10.1016/j.rmed.2011.08.018 21900000

[B31] Uchida K Nakata K Suzuki T Luisetti M Watanabe M Koch DE Stevens CA Beck DC Denson LA Carey BC **Granulocyte/macrophage-colony-stimulating factor autoantibodies and myeloid cell immune functions in healthy subjects** Blood 2009 113 2547 2556 19282464 10.1182/blood-2009-05-155689 PMC2656275

[B32] Standardized lung function testing **Official statement of the European Respiratory Society** Eur Respir J Suppl 1993 16 1 100 8499052

[B33] Horimasu Y Hattori N Ishikawa N Kawase S Tanaka S Yoshioka K Yokoyama A Kohno N Bonella F Guzman J **Different MUC1 gene polymorphisms in German and Japanese ethnicities affect serum KL-6 levels** Respir Med 2012 106 1756 1764 10.1016/j.rmed.2012.09.001 22995277

[B34] Janssen R Kruit A Grutters JC Ruven HJ Gerritsen WB van den Bosch JM **The mucin-1 568 adenosine to guanine polymorphism influences serum Krebs von den Lungen-6 levels** Am J Respir Cell Mol Biol 2006 34 496 499 10.1165/rcmb.2005-0151OC 16357367

[B35] Seymour JF Doyle IR Nakata K Presneill JJ Schoch OD Hamano E Uchida K Fisher R Dunn AR **Relationship of anti-GM-CSF antibody concentration, surfactant protein A and B levels, and serum LDH to pulmonary parameters and response to GM-CSF therapy in patients with idiopathic alveolar proteinosis** Thorax 2003 58 252 257 10.1136/thorax.58.3.252 12612307 PMC1746613

[B36] Lin FC Chang GD Chern MS Chen YC Chang SC **Clinical significance of anti-GM-CSF antibodies in idiopathic pulmonary alveolar proteinosis** Thorax 2006 61 528 534 10.1136/thx.2005.054171 16517574 PMC2111220

[B37] Hisata S Kimura Y Shibata N Ono S Kobayashi T Chiba S Ohta H Nukiwa T Ebina M **A Normal Range of KL-6/MUC1 Independent of Elevated SP-D Indicates a Better Prognosis in the Patients with Honeycombing on High-Resolution Computed Tomography** Pulm Med 2011 2011 806014 21637370 10.1155/2011/806014 PMC3101794

[B38] Ishii H Tazawa R Kaneko C Saraya T Inoue Y Hamano E Kogure Y Tomii K Terada M Takada T **Clinical features of secondary pulmonary alveolar proteinosis: pre-mortem cases in Japan** Eur Respir J 2011 37 465 468 10.1183/09031936.00092910 21282812

